# Regularity in Stock Market Indices within Turbulence Periods: The Sample Entropy Approach

**DOI:** 10.3390/e24070921

**Published:** 2022-07-01

**Authors:** Joanna Olbryś, Elżbieta Majewska

**Affiliations:** 1 Faculty of Computer Science, Bialystok University of Technology, Wiejska 45a, 15-351 Białystok, Poland; 2 Faculty of Economics and Finance, University of Bialystok, Warszawska 63, 15-062 Białystok, Poland; e.majewska@uwb.edu.pl

**Keywords:** Sample Entropy (SampEn), stock market index, regularity, predictability, Global Financial Crisis, COVID-19, rolling-window

## Abstract

The aim of this study is to assess and compare changes in regularity in the 36 European and the U.S. stock market indices within major turbulence periods. Two periods are investigated: the Global Financial Crisis in 2007–2009 and the COVID-19 pandemic outbreak in 2020–2021. The proposed research hypothesis states that entropy of an equity market index decreases during turbulence periods, which implies that regularity and predictability of a stock market index returns increase in such cases. To capture sequential regularity in daily time series of stock market indices, the Sample Entropy algorithm (SampEn) is used. Changes in the SampEn values before and during the particular turbulence period are estimated. The empirical findings are unambiguous and confirm no reason to reject the research hypothesis. Moreover, additional formal statistical analyses indicate that the SampEn results are similar both for developed and emerging European economies. Furthermore, the rolling-window procedure is utilized to assess the evolution of SampEn over time.

## 1. Introduction

The vast majority of the literature in finance relies on the informational market efficiency assumption, which implies unpredictability of financial markets. The concept of informational efficiency is central in finance and it is strictly connected with the Efficient Market Hypothesis (EMH) [1]. An efficient market is defined as one in which new information is quickly and correctly reflected in current security prices [2]. The classic taxonomy of information sets distinguishes between: (1) weak-form efficiency (the information set includes only the history of prices or returns), (2) semi-strong-form efficiency (the information set includes all public available information), and (3) strong-form efficiency (the information set includes all information known to any market participant) [3]. Although the EMH is simple in principle, it remains an elusive concept [4]. Therefore, testing for efficiency and predictability of markets is difficult, which implies that empirical results are ambiguous [2].

There is an important strand of the existing literature, known as Algorithmic Information Theory (AIT), that explores predictability in terms of sequential regularity of time series based on the existence of patterns. The AIT could be employed to investigate the regularity/irregularity in data series by analyzing its entropy [5].

Entropy was defined by Shannon as a measure of information, choice and uncertainty [6]. The concept of entropy has originated from physics (precisely, from thermodynamics), but it has been employed in various research fields to assess the information content of a probability distribution, and to describe the complexity of a system. Entropy properly characterizes the uncertainty, particularly the unpredictability, of a random variable [7]. The highest uncertainty of the system corresponds to the highest entropy. Specifically, high values of entropy are related to randomness in the evolution of stock prices [8]. In contrast, when no uncertainty exists in the system, entropy is minimized.

Entropy is an universal measure, and therefore many applications of entropy have been proposed in the literature, including economic, finance, and management studies (for a brief literature review see for instance [9,10,11,12,13,14,15,16] and the references therein).

It is important to emphasize that the fundamental mathematical entropy definitions, for instance the Kolmogorov-Sinai entropy ([17,18]), were not formulated for statistical applications. For this reason, Pincus [19] introduced the Approximate Entropy (ApEn) as a new statistic for experimental and empirical data series. The ApEn statistic was constructed along similar line to the Kolmogorov-Sinai entropy. Unfortunately, the ApEn procedure has some disadvantages which make that the results suggest more regularity than there is in reality (e.g., [5,20]).

The alternative statistic, the so-called Sample Entropy (SampEn), was proposed by Richman and Moorman [20] to avoid the ApEn bias. The SampEn algorithm solves the self-matching problem and eliminates the ApEn bias. The SampEn was initially used in physiological time series analyses, but it is also a suitable indicator for economic and financial data sets (e.g., [21,22]). Both ApEn and SampEn statistics are model-independent measures of sequential regularity in experimental or empirical data series. They are based on the existence of patterns. Moreover, they can quantify the regularity in time series with a relatively small number of data. However, due to the ApEn bias reporting in the literature, the SampEn algorithm is used in this study since it works better than the ApEn procedure (e.g., [5,20,23]).

The terms *regularity/irregularity* and *sequential regularity/irregularity* are connected with the terms *complexity* and *randomness* of data series [19]. The AIT procedure (ApEn or SampEn) assigns a nonnegative number to a sequence or time series, with larger values corresponding to greater apparent serial randomness or irregularity, and smaller values corresponding to more instances of recognizable features in the data [24]. Pincus [25] emphasizes that the need to assess potentially exploitable changes in serial structure is paramount in analyses of financial and econometric data.

The goal of this research is to assess and compare changes in sequential regularity in the 36 European and the U.S. stock market indices within major turbulence periods with the use of the SampEn statistic. Two periods are investigated: the Global Financial Crisis (GFC) in 2007–2009 and the COVID-19 pandemic outbreak in 2020–2021.

According to the literature, there is no unanimity in determining the phases of the GFC among the researchers (see, e.g., [26,27,28,29,30] and the references therein). Therefore, in our research, the GFC period was formally detected with the use of the Pagan and Sossounov [31] statistical method of dividing market states into bullish and bearish markets. The results reported in the papers [29,32,33] revealed the period from October 2007 to February 2009 as the GFC period for the U.S. and the majority of the European financial markets. The results are consistent with the literature (see, e.g., [26,27]).

The COVID-19 pandemic period comprised two years (2020–2021), since on 30 January 2020, the COVID-19 outbreak was declared as a Public Health Emergency of International Concern by the World Health Organization (WHO), while on 11 March 2020, the WHO officially declared the COVID-19 outbreak to be a global pandemic [34].

The proposed main research hypothesis states that entropy of an equity market index decreases during turbulence periods. It means that regularity and predictability of a stock market index increases within such periods. To examine the hypothesis, changes in the SampEn values for the pre-turbulence and turbulence periods are estimated.

The contribution of our study is twofold. First, the empirical findings are unambiguous and confirm no reason to reject the research hypothesis. The comparative results are especially homogenous for the pre-COVID-19 and COVID-19 sub-periods, and they support the evidence that regularity and predictability of the U.S. and almost all European stock markets indices increased during the COVID-19 outbreak. Moreover, the rolling-window approach is used to assess the evolution of entropy over time. The empirical findings are illustrated with the corresponding graphs which indicate that entropy (measured by SampEn) substantially decreased during the COVID-19 pandemic, especially in March–April 2020.

Second, the results are similar both for developed and emerging economies, and document that entropy of European developed markets does not differ significantly compared to the European emerging markets. Therefore, the findings do not support the hypothesis that developed markets are generally more efficient than emerging ones (see, e.g., [35]).

The value-added of this research derives from novel empirical findings that have not been reported in the literature thus far. These findings are important for academics and practitioners as they support the thesis that a sequential regularity in financial time series exists and even rises during extreme event periods, which implies a possibility of returns prediction. To the best of the authors’ knowledge, this is the first comparative study that investigates the group of 36 European stock markets in the context of their sequential regularity measured by SampEn.

The rest of this study is organized as follows. Section 2 presents a brief literature review. Section 3 describes the methodological background concerning the SampEn algorithm and contains data description. Section 4 presents and compares empirical results on the European stock markets and the U.S. market. The last section summarizes and discusses the main findings and indicates some further research directions. The paper is supplemented with three appendixes.

## 2. Literature Review

In light of the recently growing literature, a fairly broad research field regards assessing informational efficiency and predictability of financial markets with various entropy-based methods (e.g., [8,21,35,36,37,38,39,40,41,42,43,44,45,46]).

For instance, Zunino et al. [8] introduce and utilize two quantifiers for stock market (in)efficiency, namely the number of forbidden patterns and the normalized permutation entropy. Maasoumi and Racine [36] use a metric entropy measure of dependence to examine predictability of stock market returns. Oh et al. [37] assess efficiency of 17 foreign exchange markets using the approximate entropy approach. Risso ([35,38]) investigates informational efficiency of various stock market indices utilizing the Shannon entropy and the symbolic time series analysis. Eom et al. [39] evaluate the relationship between efficiency and predictability in 27 stock markets. They use the Hurst exponent and the approximate entropy procedure to analyse a long period of time. Gu [40] aims to predict the DJIA Index values in both short-term and long-term employing the multi-scale Shannon entropy. Ortiz-Cruz et al. [41] investigate informational complexity and efficiency of several crude oil markets with the multi-scale approximate entropy approach. Liu et al. [42] develop the conditional entropy and the transfer entropy to accommodate various trading activities in the context of market efficiency. Alvarez-Ramirez et al. [46] use entropy methods for measuring a time-varying structure of the U.S. stock market informational efficiency. Bekiros and Marcellino [43] propose a new wavelet-based approach with minimum-entropy decomposition to explore predictability of currency markets at different timescales. Gencay and Gradojevic [44] use parametric and non-parametric entropy-based methods in order to obtain an early indication of financial crisis and to predict market behavior. Wang and Wang [45] employ a multi-scale entropy-based method to analyse efficiency of various financial time series during the COVID-19 pandemic. Kim and Lee [21] use the approximate entropy, the sample entropy, and the Lempel-Ziv measure for the complexity of a time sequence to investigate predictability in cryptocurrency markets during the pandemic period. However, studies that deeply explore wide groups of stock markets in the context of their regularity and predictability are scarce.

## 3. Methodological Background and Data Description

This section presents the methodological background concerning the Sample Entropy algorithm (SampEn) and contains the real-data description.

### 3.1. The Sample Entropy Algorithm

In this research, the SampEn algorithm code in R has been implemented based on the paper [5], and therefore the similar notation has been used.

Let us consider a time sequence u={u(1),u(2),…,u(N)} of length *N*, an integer 0≤m≤N, which is the length of sequences to be compared, and a real number r>0, which denotes the tolerance for accepting matches. The parameters *N*, *m*, and *r* must be fixed for each computation.

The vectors $x_{m}\left(i\right)=\{u\left(i\right),\;u\left(i+1\right),\ldots ,$u(i + m − 1)} and $x_{m}\left(j\right)=\left\{u\left(j\right),u\left(j+1\right),\ldots ,u\left(j+m-1\right)\right\}$ are defined and then the Chebyshev distance between them is calculated based on Equation (1):

(1) $$d\left[x_{m}\left(i\right),x_{m}\left(j\right)\right]=max_{k=1,2,\ldots ,m}(|u\left(i+k-1\right)-u\left(j+k-1\right)|).$$

The number of vectors $x_{m}\left(j\right)$ within *r* of $x_{m}\left(i\right)$ without allowing self-counting is defined by Equation (2):

(2) $$B_{i}^{m}\left(r\right)=\frac{1}{N-m-1}\sum_{j=1,j\neq i}^{N-m}\left(\mathrm{number}\mathrm{of}\mathrm{times}\mathrm{that}d\left[x_{m}\left(i\right),x_{m}\left(j\right)\right]\leq r\right).$$

In the next step, the total number of possible vectors $B^{m}\left(r\right)$ is calculated based on Equation (3), and it denotes the empirical probability that two sequences match for *m* points:

(3) $$B^{m}\left(r\right)=\frac{1}{N-m}\sum_{i=1}^{N-m}B_{i}^{m}\left(r\right).$$

Analogically, the number of vectors $x_{m+1}\left(j\right)$ at a distance *r* of $x_{m+1}\left(i\right)$ without allowing self-matching is defined by Equation (4):

(4) $$A_{i}^{m}\left(r\right)=\frac{1}{N-m-1}\sum_{j=1,j\neq i}^{N-m}\left(\mathrm{number}\mathrm{of}\mathrm{times}\mathrm{that}d\left[x_{m+1}\left(i\right),x_{m+1}\left(j\right)\right]\leq r\right).$$

Next, the total number of matches $A^{m}\left(r\right)$ is computed based on Equation (5), and it denotes the empirical probability that two sequences are similar for m+1 points (matches).

(5) $$A^{m}\left(r\right)=\frac{1}{N-m}\sum_{i=1}^{N-m}A_{i}^{m}\left(r\right).$$

Since the number of matches ($A^{m}\left(r\right)$) is always less than or equal to the number of possible vectors ($B^{m}\left(r\right)$), the ratio $\frac{A^{m}\left(r\right)}{B^{m}\left(r\right)}<1$ is a conditional probability [5].

In the last step, the SampEn value of the time sequence *u* is computed as follows:

(6) $$SampEn\left(m,r,N\right)\left(u\right)=-log\left(\frac{A^{m}\left(r\right)}{B^{m}\left(r\right)}\right).$$

The SampEn(m,r,N) given by Equation (6) is the statistical estimator of the parameter SampEn(m,r):

(7) $$SampEn\left(m,r\right)=\lim_{N\to \infty }\left[-log\left(\frac{A^{m}\left(r\right)}{B^{m}\left(r\right)}\right)\right].$$

For regular, repeating data, the term $\frac{A^{m}\left(r\right)}{B^{m}\left(r\right)}$ in Equation (7) nears one, and therefore Sample Entropy nears zero [47].

### 3.2. Real-Data Description

The data set includes daily observations for the 36 European stock market indices and the S&P500 index. The sample covers the period from January, 2006 to December, 2021. The returns of stock market indices are calculated as daily logarithmic rates of return given by Equation (8):

(8) $$r_{t}=lnP_{t}-lnP_{t-1},$$

where $P_{t}$ is the daily value of the particular market index on day *t*.

Table 1 presents brief information about all analyzed indices, in order of decreasing value of stock market capitalisation in 31 December 2020, as well as the basic statistics for daily logarithmic rates of return within the whole sample period. Several results in Table 1 need comments. The sample means are not statistically different from zero. The test statistic for skewness and excess kurtosis is the conventional t-statistic. The measure for skewness indicate that almost all series are skewed at the 0.05 level of significance, except for Cyprus (*p*-value 0.287) and Bosnia and Herzegovina (*p*-value 0.894). The values of excess kurtosis show that all series are highly leptokurtic with respect to the normal distribution. Furthermore, the Jarque-Bera (J-B) test [48] rejects normality for each return series as all of the J-B statistic values are greater than 3505 with the *p*-value approximately equal to zero (these values are not reported in Table 1 but are available upon a request). It is worth noting that the obtained empirical findings are typical for return time series and are consistent with the literature (e.g., [49]). The similar Table A1 and Table A2 that report the basic statistics for daily logarithmic rates of return within the turbulence sub-periods are presented in Appendix A.

## 4. Results

This section presents empirical findings concerning sequential regularity and predictability of the 36 European stock markets and the U.S. market within the turbulence periods.

### 4.1. Empirical Experiments

In this subsection, the research hypothesis proposed in Introduction is examined. Changes in the SampEn values for the pre-turbulence and turbulence periods are estimated to assess whether entropy of equity market indices decreased during extreme event periods. To calculate the changes in entropy before and during the particular turbulence period, the following pairs of sub-periods of equal length are investigated:

1. For the Global Financial Crisis (GFC):
  - The pre-GFC period from May 2006 to September 2007 (17 months);
  - The GFC period from October 2007 to February 2009 (17 months).
2. For the COVID-19 pandemic outbreak:
  - The pre-COVID-19 pandemic period from January 2018 to December 2019 (24 months);
  - The COVID-19 pandemic period from January 2020 to December 2021 (24 months).

As was emphasized in Introduction, the aforementioned turbulence periods are based on the references [26,27,32,33,34].

An important expected feature of the SampEn algorithm is the relative consistency (e.g., [20,50]). This property follows from the Kolmogorov-Sinai definition of entropy [17]. The notion of relative consistency was introduced by Pincus [19]. In terms of the SampEn procedure, this can be written as the following property:

For dynamical processes A,B, if $SampEn\left(m_{1},r_{1}\right)\left(A\right)<SampEn\left(m_{1},r_{1}\right)\left(B\right)$, then $SampEn\left(m_{2},r_{2}\right)\left(A\right)<SampEn\left(m_{2},r_{2}\right)\left(B\right)$.

This property means that if series *A* exhibits more sequential regularity than series *B* for one set of the parameters $\left(m_{1},r_{1}\right)$, then this holds true for any other set $\left(m_{2},r_{2}\right)$ [20]. This expected property enables us to compare two processes for a single set $\left(m_{1},r_{1}\right)$ and draw conclusions for all sets of input parameters.

As mentioned in Section 3.1, the SampEn statistic depends on three parameters: *N*, *m*, and *r*, where *N* is a time series length, *m* is the length of sequences to be compared, and a real number r>0 denotes the tolerance for accepting matches. Based on the literature, the suggestion is that *m* should be 1 or 2, since there are more template matches for m=1, but m=2 (or greater) reveals more of the dynamics of the data. Moreover, the authors of the SampEn procedure suggest that *r* should be 0.2 times the standard deviation σ of the empirical data set [47]. Therefore, in this research, the m=2 and r=0.2σ parameters are used.

Table 2 includes the SampEn empirical findings within the Global Financial Crisis and COVID-19 pandemic outbreak. The columns entitled ‘Change’ report changes in entropy before and during particular turbulence period. The down arrows show entropy decrease, while the (rare) up arrows visualize entropy increase.

The results presented in Table 2 require some explanations and interpretations. In general, the empirical findings are unambiguous and confirm no reason to reject the research hypothesis. The evidence is that entropy decreased within the GFC period for the U.S. and the vast majority of the European markets, except for nine countries (i.e., Switzerland, Sweden, Finland, Ireland, Serbia, Malta, Cyprus, Estonia, Bosnia and Herzegovina). Both developed and emerging markets are among them. The probable reason of the differences in the obtained results is that the GFC periods for some countries were slightly different (for details see, e.g., [33]).

However, the comparative results for the pre-COVID-19 and COVID-19 sub-periods are homogenous and they decidedly support the evidence that regularity and predictability of the U.S. and almost all European stock markets (apart from Ukraine) increased during the COVID-19 outbreak. Due to the investigated period (2020-2021), the isolated case of Ukraine is rather coincidental and is not connected with the Russian aggression in Ukraine on 24 February 2022.

The ranges of the SampEn values for the European market indices are: [1.478;2.118] (Pre-GFC), [0.554;2.078] (GFC), [0.730;2.253] (Pre-COVID), and [0.566;2.001] (COVID). The minimum, maximum, median, and mean values decreased substantially during both extreme event periods.

To formally test whether the mean results of SampEn for the whole group of markets during the turbulence period differ significantly compared to the corresponding pre-turbulence period, the *t* statistic for sample means given by Equation (9) is utilized:

(9) $$t=\frac{\left(\bar{x_{1}}-\bar{x_{2}}\right)\sqrt{n}}{\sqrt{s_{1}^{2}+s_{2}^{2}}},$$

where $\bar{x_{1}}$ and $\bar{x_{2}}$ are sample means, $s_{1}^{2}$ and $s_{2}^{2}$ are sample variances, while n=36 denotes the stock markets sample size.

The following two-tailed hypothesis is tested:

(10) $$\begin{array}{c} H_{0}:\mu_{1}=\mu_{2}\\ H_{1}:\mu_{1}\neq \mu_{2}, \end{array}$$

where $\mu_{1}$ and $\mu_{2}$ are the expected values of SampEn for the whole group of stock market indices during the compared periods, and the null hypothesis states that two expected values are equal. Calculations of the *t* statistic values (Equation (9)) are based on the results presented in Table 2. The null hypothesis is rejected when $|t|>t^{*}$, where the critical value of *t*-statistic at the α significance level is equal to $t^{*}=t_{\alpha ;2n-2}$. In our research, the critical values are equal to: $t^{*}=1.667$ (α=0.10), $t^{*}=1.994$ (α=0.05), and $t^{*}=2.648$ (α=0.01), respectively.

The obtained empirical *t*-statistics are equal to: (1) $t=3.325>t^{*}$ for the pair of periods (pre-GFC, GFC), and (2) $t=5.036>t^{*}$ for the pair of periods (pre-COVID, COVID). This indicates that the $H_{0}$ hypothesis was rejected in both cases and the SampEn mean values substantially differed (specifically, decreased) during both extreme event periods.

What is important, the SampEn findings are consistent with the literature as they confirm that entropy of stock market indices usually decreases during the economic downturns (see, e.g., [41,45]). The equity market crash initiates a declining trend, which reduces entropy but increases time series regularity. As a consequence, predictability of a market increases within turbulence periods since a number of repeated patterns increases. It is worth noting that this evidence is in accordance with investors’ intuition.

### 4.2. Sample Entropy of Developed versus Emerging European Stock Markets

An interesting and important question is whether developed stock markets differ substantially from emerging markets in their predictability, in the sense of their sequential regularity. Therefore, in this subsection, the comparative assessment of regularity/irregularity in the European developed and emerging markets is presented.

Based on the recent MSCI reports, and especially on the report “MSCI Global Market Accessibility Review. Country comparison” [51], the following 15 European countries are classified as developed markets (in the order of decreasing value of stock market capitalisation in 31 December 2020 reported in Table 1): France, United Kingdom, Germany, Switzerland, Netherlands, Sweden, Spain, Italy, Denmark, Belgium, Finland, Norway, Ireland, Austria, and Portugal. The remaining 21 European countries are recognized as emerging, including also frontier and stand-alone equity markets (see Table A3 in Appendix B).

**Figure 1:**
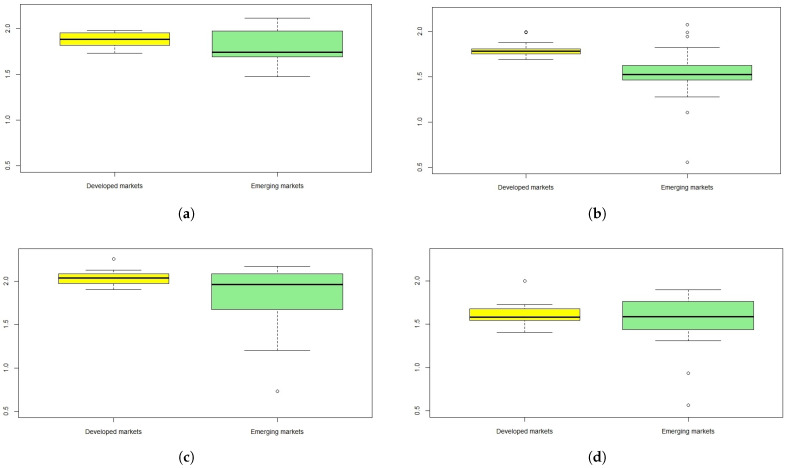
The boxplots of the SampEn results for the European developed (the yellow boxplots) and emerging (the green boxplots) countries: (**a**) the SampEn within the pre-GFC period, (**b**) the SampEn within the GFC period, (**c**) the SampEn within the pre-COVID-19 pandemic period, (**d**) the SampEn within the COVID-19 pandemic period.

Figure 1 presents the boxplots of the SampEn results within the pre-turbulence and turbulence periods, for two groups of the European developed (the yellow boxplots) and emerging (the green boxplots) stock markets. The boxplots that visualize the SampEn results are based on Table A4 and Table A5 (Appendix B). The boxplot width depends on the number of the stock market indices, and these numbers are: 15 (the European developed markets) and 21 (the European emerging markets).

One can observe that entropy measured by the SampEn statistic substantially fell during the turbulence periods compared to the pre-turbulence periods, respectively. The down arrows in Table A4 and Table A5 illustrate the substantial falls of median and percentile values.

The SampEn median for the European developed markets was equal to 1.88 (within the pre-GFC period) versus 1.79 (within the GFC period). Similarly, the corresponding SampEn median values for the European emerging markets were equal to: 1.74 (within the pre-GFC period) and 1.53 (within the GFC period), respectively (see Table A4).

As for the pre-COVID-19 and COVID-19 sub-periods, the changes in entropy were even more significant. For the European developed markets the SampEn median values were equal to 2.04 versus 1.58, while for the European emerging markets, 1.96 versus 1.59 (see Table A5).

To formally test the hypothesis concerning the median values within pre-turbulence and turbulence periods, the following conditions are proposed:

(11) $$\begin{array}{c} H_{0}:Me_{1}=Me_{2}\\ H_{1}:Me_{1}>Me_{2}, \end{array}$$

where $Me_{1}$ is a SampEn median value before particular turbulence period, while $Me_{2}$ denotes a SampEn median value during a turbulence period, respectively. The null hypothesis states that two median values are equal. To examine the hypothesis, the Wilcoxon-Mann-Whitney test [52] is used and the calculations are reported in Table 3. The numbers in brackets are *p*-values. The test results indicate that the null hypothesis $H_{0}$ should be rejected in all cases, both for developed and emerging markets. Hence, the evidence is that the median values during the turbulence periods were significantly lower compared to the corresponding pre-turbulence periods.

The boxplot height means the interquartile range, which is a measure of statistical dispersion as it is equal to the difference between Q3 (75th) and Q1 (25th) percentiles. The evidence is that the level of entropy dispersion for the European developed market indices was similar and low, regardless of the time period choice. The results for the European emerging markets are mixed, but the main probable reason is that these markets are much more diverse. However, one can observe that the interquartile range for the emerging markets has substantially decreased during the turbulence periods (see Table A4 and Table A5).

The singular points denote outliers. The SampEn outliers were: (1) within the GFC period: Switzerland, Finland, Turkey, Poland, Cyprus (significantly higher values of the SampEn) and Slovakia (significantly lower value of the SampEn), (2) within the pre-pandemic period: Finland (significantly higher value of the SampEn) and Bosnia and Herzegovina (significantly lower value of the SampEn), and (3) within the pandemic period: Denmark (significantly higher value of the SampEn) and Slovakia and Bosnia and Herzegovina (significantly lower values of the SampEn). Within the pre-GFC period outliers did not appear.

To formally test the hypothesis concerning the comparison between the SampEn median values of the European developed and emerging stock markets within various sub-periods, the following $H_{0}$ and $H_{1}$ conditions (Equation (12)) are proposed:

(12) $$\begin{array}{c} H_{0}:Me_{1}=Me_{2}\\ H_{1}:Me_{1}\neq Me_{2}, \end{array}$$

where $Me_{1}$ is a SampEn median value of the group of the European developed markets, while $Me_{2}$ denotes a SampEn median value of the group of the European emerging markets, respectively. The null hypothesis states that two median values are equal. To examine the hypothesis, the Wilcoxon-Mann-Whitney test [52] for two independent groups is used, and the calculations are reported in Table 4. The numbers in brackets are *p*-values. The test results indicate that the null hypothesis $H_{0}$ should be rejected only during the GFC period (*p*-value 0.0019), while there is no reason to reject the null hypothesis for other periods. Therefore, the evidence is that the SampEn median values did not differ significantly between developed and emerging markets during the remaining three sub-periods.

To summarize, the findings for both the European developed and emerging equity markets are homogenous. The analyzed groups of market indices do not differ substantially in their sequential regularity. The aforementioned SampEn results indicate that entropy visibly fell during each extreme event period compared to the corresponding pre-event period. It implies that predictability of market indices rose, which confirmed no reason to reject the main research hypothesis.

### 4.3. The Evolution of Sample Entropy over Time

In this subsection, the evolution of SampEn over time is analyzed. A rolling-window dynamic approach is employed to capture the changes in market index regularity (measured by SampEn) through time, for daily logarithmic index returns.

In line of the existing literature, the sample size *N* should be within the range of $\left[10^{m},30^{m}\right]$ (see, e.g., [5,45]). As pointed out in Section 4.1, in this research m=2, hence the minimal time window length should be equal to 100. Therefore, a window N=100 business days is utilized in this study.

The broad group of 36 stock markets is explored. The use of the rolling-window method requires the corresponding figures that show the changes in SampEn over time. Hence, it should be 36 × 2 = 72 figures reported in the paper as the graphic representation of the rolling-window procedure. Therefore, only selected dynamic SampEn results for developed and emerging markets are illustrated, i.e., the results for stock markets with the highest absolute value of the change in SampEn (based on Table 2). Due to the space restriction, the remaining figures are available upon a request.

**Figure 2:**
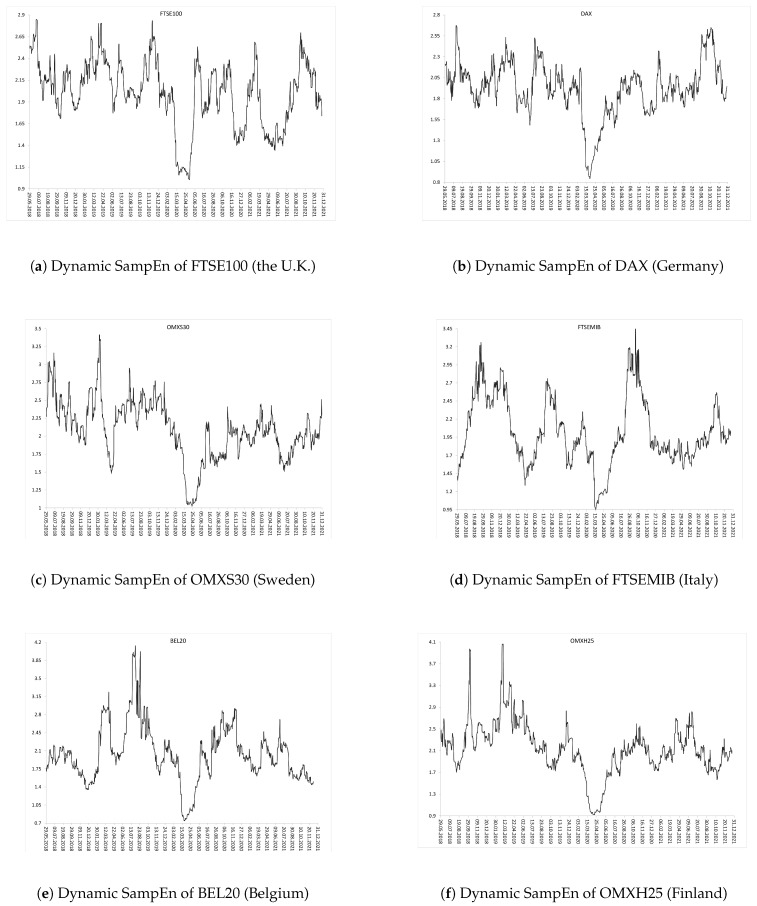
Dynamic SampEn of the selected European developed market indices within the period from January 2018 to December 2021 (two combined pre-COVID-19 and COVID-19 sub-periods): (**a**) FTSE100 (the U.K.), (**b**) DAX (Germany), (**c**) OMXS30 (Sweden), (**d**) FTSEMIB (Italy), (**e**) BEL20 (Belgium), (**f**) OMXH25 (Finland). The rolling-window N=100 business days.

**Figure 3:**
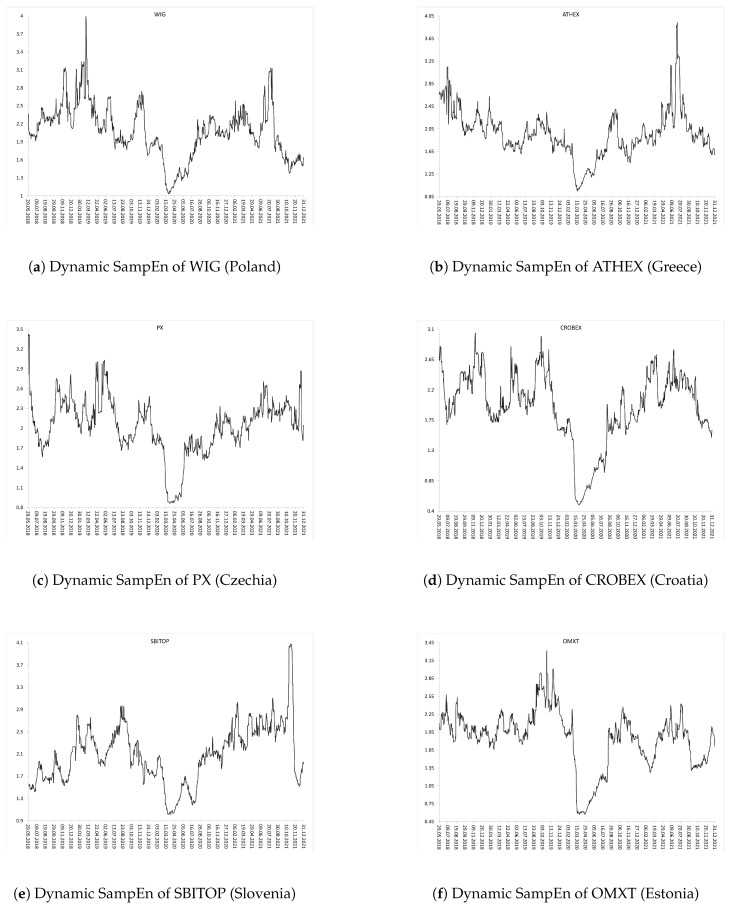
Dynamic SampEn of the selected European emerging market indices within the period from January 2018 to December 2021 (two combined pre-COVID-19 and COVID-19 sub-periods): (**a**) WIG (Poland), (**b**) ATHEX (Greece), (**c**) PX (Czechia), (**d**) CROBEX (Croatia), (**e**) SBITOP (Slovenia), (**f**) OMXT (Estonia). The rolling-window N=100 business days.

**Figure 4:**
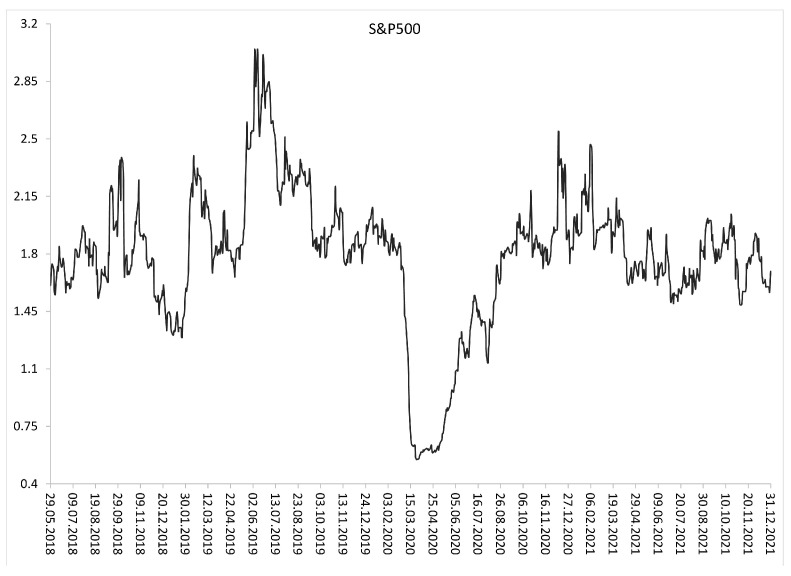
Dynamic SampEn of the S&P500 index (the U.S.) within the period from January 2018 to December 2021 (two combined pre-COVID-19 and COVID-19 periods). The rolling-window N=100 business days.

Subsequent Figure 2, Figure 3 and Figure 4 show the evolution of SampEn over time within the period from January 2018 to December 2021 (two combined pre-COVID-19 and COVID-19 sub-periods). Figure 2 and Figure 3 present graphs for the European developed and emerging markets, respectively. Finally, Figure 4 plots the dynamics of SampEn for S&P500 index. The SampEn procedure implemented on the rolling-window scheme indicates and confirms that entropy visibly decreased during the COVID-19 pandemic, especially in March-April 2020, both for developed and emerging markets. It is rather clear that the main reason of such homogenous results is that all investigated stock markets have been affected by the COVID-19 pandemic in the same time and to the similar extent.

By analogy, the rolling-window procedure is utilized to investigate the evolution of the SampEn during the period from May 2006 to February 2009 (two combined pre-GFC and GFC sub-periods). The findings are reported and discussed in Appendix C.

## 5. Discussion and Conclusions

The goal of this empirical study was to investigate changes in sequential regularity in the 36 European and the U.S. stock market indices within major turbulence periods. Two periods were analyzed: the Global Financial Crisis in 2007–2009 and the COVID-19 pandemic outbreak in 2020–2021. To capture regularity in the daily time series of stock market indices, the SampEn algorithm was utilized. Changes in the SampEn values before and during the particular turbulence period were calculated and compared. The research hypothesis that entropy of an equity market index decreases during turbulence periods was examined. The main contribution of this research lies in important empirical findings which indicate no reason to reject the research hypothesis. Our research belongs to the strand of the literature known as Algorithmic Information Theory (AIT). The AIT explores predictability in terms of sequential regularity in various time series based on the existence of patterns.

The obtained results are homogenous and statistically significant for both investigated turbulence periods, and for both independent groups of stock markets (developed and emerging). The findings imply that regularity in stock market index returns increases during extreme event periods.

Our results contribute to the discussion concerning predictability of financial markets. It seems that the conclusions could be generalized as the SampEn empirical findings are in line with the relatively scarce previous literature which documents that entropy of various financial time series usually decreases during market crashes, financial crisis and other turbulence periods. For instance, Ortiz-Cruz et al. [41] utilized the multi-scale approximate entropy procedure and they indicated that returns from crude oil markets were less uncertain during economic downturns. Wang and Wang [45] assessed informational efficiency of S&P500 Index, gold, Bitcoin, and US Dollar Index during the COVID-19 pandemic with a multi-scale entropy-based method. They confirmed that a decline of entropy was particularly large for S&P500 Index. Moreover, their results of dynamic informational efficiency of the S&P500 Index are similar to ours. Risso [38] investigated several market indices during financial crashes. He showed that short-time market trends (both ‘up’ and ‘down’) usually reduce entropy of an index daily time series due to more frequent patterns.

Moreover, it is worth noting that, during the turbulence periods, all public information is especially important for investors and determines investment decisions. However, the used information set includes only the history of index returns. Therefore, our research relates to the literature concerning the weak form of market informational efficiency. The obtained results indicate that informational efficiency of stock market indices decreases during turbulence periods. This evidence is especially useful for investors as it provides information about a possibility of financial forecasting.

The findings of our research might be interesting for academics and practitioners since the entropy-based indicators can generate predictive signals and can be useful in predictive modelling (see, e.g., [44,53]). Moreover, there are some innovative applications of entropy for financial time series forecasting (see, e.g., [43,54]). Gradojevic and Caric [54] emphasize that although volatility and entropy are related measures of market risk and uncertainty, entropy can be more useful in predictive modelling. Taking the above into consideration, we hope that the results of our research could be generally of special importance for investors as the entropy-based procedures might be used as helpful tools in various systems that support investment decisions.

Since the analyzed GFC and COVID-19 periods have affected all financial markets in the world, the promising direction for further research could be an extensive comparative assessment of predictability in the context of sequential regularity in time series of stock market indices within the world, for instance in continent-based regions. Moreover, the influence of the recent extreme event, i.e., the Russian invasion in Ukraine, could be investigated.

## Figures and Tables

**Table 1:** The information about the analyzed stock market indices and the basic statistics for daily logarithmic rates of return within the whole sample period.

	Country	Index	Market Cap.	Mean	Std. Dev.	Skewness	Excess
			EUR Billion	(in %)	(in %)		Kurtosis
			Dec 2020				
	United States	S&P500	18,435.290	0.0329	1.26	−0.567	13.739
1	France	CAC40	2480.404	0.0100	1.39	−0.290	8.324
2	United Kingdom	FTSE100	2411.490	0.0065	1.18	−0.390	9.829
3	Germany	DAX	1870.687	0.0264	1.37	−0.239	8.257
4	Switzerland	SMI	1639.314	0.0130	1.11	−0.418	9.700
5	Netherlands	AEX	1149.619	0.0145	1.29	−0.396	9.549
6	Sweden	OMXS30	873.404	0.0229	1.37	−0.200	5.724
7	Spain	IBEX35	621.765	−0.0052	1.48	−0.378	9.717
8	Italy	FTSEMIB	600.652	−0.0067	1.59	−0.679	9.848
9	Russia	RTSI	568.992	0.0073	2.09	−0.574	11.988
10	Denmark	OMXC20	506.525	0.0385	1.28	−0.352	5.983
11	Belgium	BEL20	306.132	0.0046	1.27	−0.666	10.853
12	Finland	OMXH25	289.000	0.0105	1.34	−0.272	5.332
13	Norway	OSEAX	273.141	0.0309	1.42	−0.705	7.149
14	Turkey	XU100	194.491	0.0383	1.63	−0.467	4.479
15	Poland	WIG	145.379	0.0163	1.25	−0.746	7.141
16	Ireland	ISEQ	138.719	0.0033	1.47	−0.713	8.157
17	Austria	ATX	108.176	0.0012	1.59	−0.515	8.205
18	Portugal	PSI20	73.361	−0.0106	1.25	−0.387	7.323
19	Greece	ATHEX	41.758	−0.0356	2.00	−0.478	7.140
20	Hungary	BUX	22.908	0.0221	1.50	−0.274	8.436
21	Czechia	PX	21.797	−0.0010	1.34	−0.628	17.455
22	Romania	BET	20.895	0.0162	1.42	−0.749	12.300
23	Croatia	CROBEX	18.206	0.0010	1.10	−0.502	23.966
24	Bulgaria	SOFIX	14.505	−0.0065	1.11	−1.253	15.194
25	Lithuania	OMXV	12.114	0.0192	0.99	−0.749	26.775
26	Iceland	OMXI	9.752	−0.0171	2.06	−36.153	1806.18
27	Slovenia	SBITOP	6.919	0.0073	1.04	−0.716	10.209
28	Serbia	BELEXLINE	4.437	−0.0032	0.81	0.156	16.365
29	Malta	MSE	4.161	−0.0058	0.67	0.141	8.658
30	Cyprus	GENERAL	3.844	−0.0820	2.28	0.042	7.862
31	Ukraine	UX	3.615	0.0186	1.88	−0.283	9.630
32	Montenegro	MONEX	3.178	0.0001	1.25	0.733	13.045
33	Estonia	OMXT	3.014	0.0275	1.04	−0.412	14.786
34	Latvia	OMXR	2.971	0.0152	1.24	0.080	18.658
35	Bosnia and Herzegovina	BIFX	2.698	−0.0369	0.86	0.005	9.535
36	Slovakia	SAX	2.648	−0.0006	1.11	−0.959	21.294

**Table 2:** The SampEn empirical findings within the Global Financial Crisis and COVID-19 pandemic outbreak.

		SampEn			SampEn		
	Stock Market	Pre-GFC	GFC	Change	Pre-COVID-19	COVID-19	Change
	United States	1.798	1.734	−0.064 ↓	1.801	1.305	−0.496 ↓
1	France	1.962	1.772	−0.189 ↓	1.972	1.489	−0.482 ↓
2	United Kingdom	1.897	1.878	−0.019 ↓	2.107	1.481	−0.626 ↓
3	Germany	1.900	1.786	−0.115 ↓	1.970	1.405	−0.565 ↓
4	Switzerland	1.967	1.993	0.025 ↑	2.010	1.577	−0.432 ↓
5	Netherlands	1.950	1.773	−0.177 ↓	1.945	1.561	−0.384 ↓
6	Sweden	1.778	1.787	0.010 ↑	2.126	1.624	−0.502 ↓
7	Spain	1.884	1.796	−0.088 ↓	1.901	1.673	−0.228 ↓
8	Italy	1.850	1.695	−0.155 ↓	2.037	1.543	−0.494 ↓
9	Russia	1.803	1.278	−0.525 ↓	2.103	1.762	−0.341 ↓
10	Denmark	1.871	1.766	−0.104 ↓	2.010	2.001	−0.009 ↓
11	Belgium	1.980	1.812	−0.168 ↓	2.073	1.547	−0.526 ↓
12	Finland	1.825	1.994	0.169 ↑	2.253	1.680	−0.573 ↓
13	Norway	1.964	1.744	−0.221 ↓	2.078	1.660	−0.419 ↓
14	Turkey	2.074	1.945	−0.129 ↓	2.172	1.789	−0.383 ↓
15	Poland	2.054	1.991	−0.063 ↓	2.121	1.680	−0.442 ↓
16	Ireland	1.735	1.811	0.075 ↑	2.089	1.699	−0.390 ↓
17	Austria	1.817	1.728	−0.090 ↓	1.950	1.584	−0.366 ↓
18	Portugal	1.787	1.700	−0.086 ↓	2.069	1.728	−0.341 ↓
19	Greece	1.904	1.607	−0.297 ↓	2.084	1.559	−0.524 ↓
20	Hungary	2.118	1.525	−0.593 ↓	2.124	1.823	−0.301 ↓
21	Czechia	1.855	1.511	−0.344 ↓	2.037	1.515	−0.522 ↓
22	Romania	2.034	1.827	−0.207 ↓	1.672	1.498	−0.173 ↓
23	Croatia	2.053	1.505	−0.547 ↓	2.079	1.310	−0.769 ↓
24	Bulgaria	1.730	1.499	−0.231 ↓	1.961	1.647	−0.314 ↓
25	Lithuania	1.764	1.530	−0.234 ↓	1.520	1.408	−0.112 ↓
26	Iceland	1.743	0.554	−1.189 ↓	2.044	1.825	−0.219 ↓
27	Slovenia	1.693	1.386	−0.307 ↓	2.105	1.740	−0.364 ↓
28	Serbia	1.508	1.570	0.061 ↑	1.949	1.585	−0.364 ↓
29	Malta	1.478	1.531	0.053 ↑	1.936	1.701	−0.236 ↓
30	Cyprus	1.739	2.078	0.339 ↑	1.979	1.898	−0.081 ↓
31	Ukraine	1.486	1.466	−0.020 ↓	1.201	1.858	0.658 ↑
32	Montenegro	1.740	1.480	−0.260 ↓	1.832	1.437	−0.395 ↓
33	Estonia	1.600	1.627	0.027 ↑	1.811	1.403	−0.408 ↓
34	Latvia	1.974	1.467	−0.507 ↓	1.584	1.496	−0.089 ↓
35	Bosnia and Herzegovina	1.701	1.800	0.099 ↑	0.730	0.566	−0.164 ↓
36	Slovakia	1.641	1.106	−0.534 ↓	1.247	0.934	−0.313 ↓
	Max	2.118	2.078	−0.040 ↓	2.253	2.001	−0.253 ↓
	Min	1.478	0.554	−0.924 ↓	0.730	0.566	−0.164 ↓
	Median	1.838	1.714	−0.124 ↓	2.010	1.584	−0.426 ↓
	Mean	1.829	1.648	−0.182 ↓	1.913	1.575	−0.339 ↓
	Std. Dev.	0.164	0.284	0.120 ↑	0.310	0.258	−0.052 ↓

**Table 3:** The comparison of SampEn median values between pre-turbulence and turbulence periods—the Wilcoxon-Mann-Whitney test summary.

	Pre-GFC vs. GFC	Pre-COVID vs. COVID
European developed markets	170 (0.0082)	220 (0.0000)
European emerging markets	337 (0.0014)	345 (0.0007)

The numbers in brackets are *p*-values.

**Table 4:** The comparison of SampEn median values between the European developed and emerging stock markets. The Wilcoxon-Mann-Whitney test results.

	European Developed vs. Emerging Stock Markets
Pre-GFC period	203 (0.1504)
GFC period	252 (0.0019)
Pre-COVID period	202.5 (0.1533)
COVID period	160 (0.9495)

The numbers in brackets are *p*-values.

**Figure A1:**
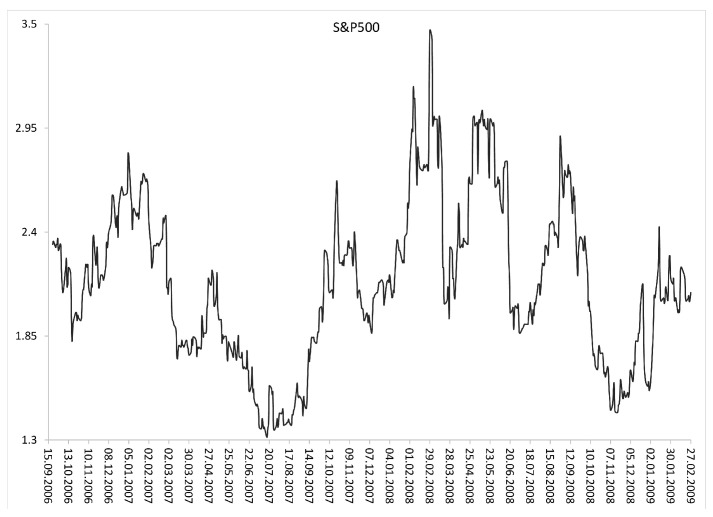
Dynamic SampEn of the S&P500 index (the U.S.) within the period from May 2006 to February 2009 (two combined pre-GFC and GFC sub-periods). The rolling-window N=100 business days.

**Figure A2:**
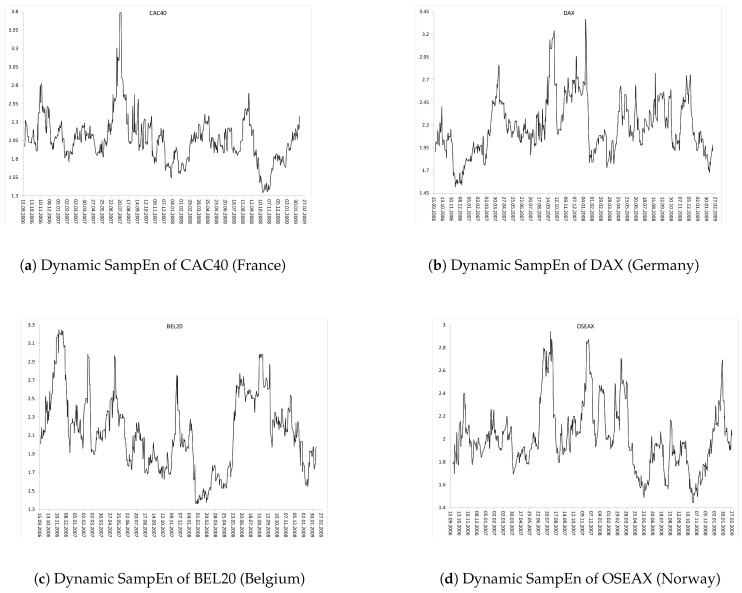
Dynamic SampEn of the selected European developed market indices within the period from May 2006 to February 2009 (two combined pre-GFC and GFC sub-periods): (**a**) CAC40 (France), (**b**) DAX (Germany), (**c**) BEL20 (Belgium), (**d**) OSEAX (Norway). The rolling-window N=100 business days.

**Figure A3:**
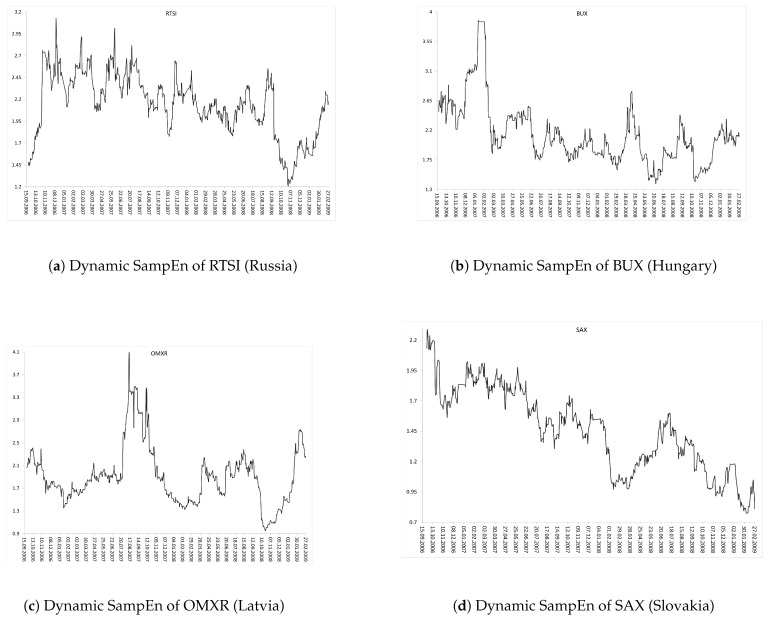
Dynamic SampEn of the selected European emerging market indices within the period from May 2006 to February 2009 (two combined pre-GFC and GFC sub-periods): (**a**) RTSI (Russia), (**b**) BUX (Hungary), (**c**) OMXR (Latvia)), (**d**) SAX (Slovakia). The rolling-window N=100 business days.

## Data Availability

The data comes from the following web pages: Stooq (https://stooq.pl, 15 January 2022); Yahoo (https://finance.yahoo.com, 15 January 2022); Malta Stock Exchange (https://www.borzamalta.com.mt, 15 January 2022); Cyprus Stock Exchange (http://www.cse.com.cy, 15 January 2022); Nasdaq (http://www.nasdaqomxnordic.com, 15 January 2022); Zagreb Stock Exchange (http://zse.hr, 15 January 2022); Ljubljana Stock Exchange (http://www.ljse.si, 15 January 2022); Montenegro Stock Exchange (http://mnse.me, 15 January 2022); Belgrade Stock Exchange (http://www.belex.rs, 15 January 2022); The Sarajevo Stock Exchange (http://www.sase.ba, 15 January 2022).

## Abbreviations

The following abbreviations are used in this manuscript:

AIT	Algorithmic Information Theory
ApEn	Approximate Entropy
SampEn	Sample Entropy
GFC	Global Financial Crisis
COVID-19	COVID-19 pandemic

## Appendix A. Basic Statistics for Sub-Periods

Table A1 and Table A2 report the basic statistics for daily logarithmic rates of return within the turbulence sub-periods. *N* denotes the number of observations. The stock markets are presented in the same order as in Table 1.

**Table A1:** The basic statistics for daily logarithmic rates of return within the pre-GFC and GFC periods.

		Pre-GFC			GFC		
	Country	N	Mean	Std. Dev.	N	Mean	Std. Dev.
			(in %)	(in %)		(in %)	(in %)
	United States	356	0.044	0.80	355	−0.210	2.37
1	France	362	0.024	1.05	360	−0.211	2.29
2	United Kingdom	358	0.017	0.97	358	−0.148	2.13
3	Germany	361	0.072	1.02	356	−0.203	2.17
4	Switzerland	355	0.028	0.93	351	−0.186	1.98
5	Netherlands	362	0.039	0.97	360	−0.253	2.39
6	Sweden	356	0.044	1.33	353	−0.185	2.36
7	Spain	362	0.055	1.02	356	−0.183	2.24
8	Italy	360	0.012	0.91	355	−0.274	2.17
9	Russia	354	0.053	1.79	346	−0.383	3.78
10	Denmark	356	0.063	1.07	351	−0.212	2.24
11	Belgium	362	0.026	0.97	361	−0.275	2.12
12	Finland	357	0.071	1.17	353	−0.290	2.20
13	Norway	356	0.053	1.45	354	−0.226	2.69
14	Turkey	359	0.057	1.87	353	−0.230	2.53
15	Poland	355	0.089	1.37	351	−0.291	1.91
16	Ireland	360	−0.001	1.20	358	−0.376	2.78
17	Austria	350	0.020	1.36	348	−0.323	2.81
18	Portugal	362	0.049	0.74	360	−0.196	1.84
19	Greece	357	0.055	1.14	350	−0.345	2.25
20	Hungary	355	0.041	1.38	348	−0.296	2.51
21	Czechia	354	0.058	1.25	354	−0.296	2.71
22	Romania	353	0.071	1.38	349	−0.469	2.64
23	Croatia	354	0.197	0.93	347	−0.374	2.39
24	Bulgaria	353	0.209	0.87	345	−0.565	2.25
25	Lithuania	346	0.093	0.93	340	−0.373	1.85
26	Iceland	353	0.105	0.94	350	−0.789	6.18
27	Slovenia	350	0.239	1.00	349	−0.319	1.99
28	Serbia	357	0.214	0.83	357	−0.432	1.47
29	Malta	350	−0.060	0.77	346	−0.159	0.68
30	Cyprus	354	0.165	1.52	344	−0.555	3.03
31	Ukraine	343	0.250	1.67	347	−0.475	2.82
32	Montenegro	348	0.377	1.66	345	−0.425	2.66
33	Estonia	358	0.085	1.02	352	−0.341	1.60
34	Latvia	354	0.051	0.80	348	−0.357	1.90
35	Bosnia and Herzegovina	357	0.186	1.42	349	−0.428	1.42
36	Slovakia	344	0.012	0.72	343	−0.077	0.95

**Table A2:** The basic statistics for daily logarithmic rates of return within the pre-COVID and COVID periods.

		Pre-COVID-19			COVID-19		
	Country	N	Mean	Std. Dev.	N	Mean	Std. Dev.
			(in %)	(in %)		(in %)	(in %)
	United States	502	0.036	0.94	504	0.075	1.65
1	France	509	0.024	0.86	514	0.033	1.58
2	United Kingdom	504	−0.003	0.77	506	−0.006	1.43
3	Germany	501	0.006	0.94	508	0.034	1.61
4	Switzerland	497	0.023	0.80	505	0.037	1.17
5	Netherlands	509	0.020	0.80	514	0.051	1.42
6	Sweden	499	0.023	0.92	504	0.058	1.44
7	Spain	509	−0.011	0.82	512	−0.021	1.69
8	Italy	503	0.015	1.05	510	0.027	1.74
9	Russia	504	0.053	1.28	504	0.004	2.05
10	Denmark	495	0.021	0.94	500	0.099	1.27
11	Belgium	509	−0.001	0.85	514	0.014	1.61
12	Finland	499	0.007	0.86	497	0.045	1.38
13	Norway	497	0.026	0.90	503	0.045	1.41
14	Turkey	499	−0.005	1.35	500	0.094	1.66
15	Poland	494	−0.020	0.89	502	0.032	1.51
16	Ireland	505	0.004	0.93	509	0.029	1.59
17	Austria	497	−0.016	0.95	505	0.0356	1.82
18	Portugal	509	−0.009	0.78	514	0.011	1.38
19	Greece	495	0.023	1.23	497	−0.008	2.01
20	Hungary	489	0.032	0.98	502	0.018	1.52
21	Czechia	498	0.006	0.62	500	0.048	1.24
22	Romania	497	0.047	1.04	500	0.055	1.23
23	Croatia	493	0.019	0.46	499	0.005	1.08
24	Bulgaria	491	−0.038	0.58	492	0.023	1.01
25	Lithuania	495	0.016	0.57	498	0.060	0.86
26	Iceland	494	0.033	0.80	498	0.108	1.16
27	Slovenia	490	0.031	0.55	503	0.060	1.05
28	Serbia	502	0.009	0.46	502	−0.001	0.56
29	Malta	493	0.009	0.47	493	−0.034	0.82
30	Cyprus	489	−0.012	0.83	492	0.010	1.05
31	Ukraine	488	0.024	0.95	493	0.027	1.38
32	Montenegro	493	0.024	0.63	498	−0.029	0.72
33	Estonia	500	0.004	0.45	500	0.090	1.18
34	Latvia	494	0.007	1.07	496	0.041	1.27
35	Bosnia and Herzegovina	496	0.034	0.78	502	0.004	0.37
36	Slovakia	482	0.016	0.95	490	0.026	1.07

## Appendix B. Developed and Emerging Markets

Table A3 presents two groups of the investigated stock markets: (1) the European developed markets and (2) the European emerging markets. This division is the recent one since it is based on the report “MSCI Global Market Accessibility Review. Country comparison” [51].

Table A4 and Table A5 report the SampEn basic statistics within the pre-turbulence and turbulence periods, respectively. Two groups of the European countries (presented in Table A3) are explored.

**Table A3:** The European developed and emerging stock markets.

European Developed Markets	European Emerging Markets
France, U.K., Germany, Switzerland,	Russia, Turkey, Poland, Greece,
Netherlands, Sweden, Spain, Italy,	Hungary, Czechia, Romania, Croatia,
Denmark, Belgium, Finland, Norway,	Bulgaria, Lithuania, Iceland, Slovenia,
Ireland, Austria, Portugal	Serbia, Malta, Cyprus, Ukraine,
	Montenegro, Estonia, Latvia,
	Bosnia and Herzegovina, Slovakia

Based on the MSCI report [51], in the market order as in Table 1.

**Table A4:** The SampEn basic statistics within the pre-GFC and GFC periods for developed and emerging European stock markets.

	Pre-GFC					GFC				
	Min	Max	Median	Q1	Q3	Min	Max	Median	Q1	Q3
Developed	1.74	1.98	1.88	1.82	1.96	1.69	1.99	1.79 ↓	1.76 ↓	1.81 ↓
markets										
Emerging	1.48	2.12	1.74	1.69	1.97	0.55	2.08	1.53 ↓	1.47 ↓	1.63 ↓
markets										

**Table A5:** The SampEn basic statistics within the pre-COVID-19 and COVID-19 periods for developed and emerging European stock markets.

	Pre-COVID-19					COVID-19				
	Min	Max	Median	Q1	Q3	Min	Max	Median	Q1	Q3
Developed	1.90	2.25	2.04	1.97	2.08	1.41	2.00	1.58 ↓	1.54 ↓	1.68 ↓
markets										
Emerging	0.73	2.17	1.96	1.67	2.08	0.57	1.90	1.59 ↓	1.44 ↓	1.76 ↓
markets										

## Appendix C. Dynamic SampEn Results during the Period from May 2006 to February 2009

According to the literature, the financial crisis timeline, from the U.S. perspective, was marked by the following events: (1) the increase in subprime delinquency rates in the spring of 2007, (2) the ensuing liquidity crunch in late 2007, (3) the liquidation of Bear Stearns in March 2008, and (4) the failure of Lehman Brothers in September 2008. The U.S. economy officially slipped into a recession following the peak in December 2007. It is important to note that the crisis began in the U.S., but initially it did not fully and strongly affect all financial markets. For instance, Claessens et al. [30] identify five groups of countries based on the date they were affected by the crisis. Hence, the investigated stock markets were not affected by the GFC in the same time and to the same extent. This is the probable reason why empirical findings of the SampEn dynamics for various countries are ambiguous.

Figure A1 shows the dynamics of SampEn of the S&P500 index. Moreover, Figure A2 and Figure A3 illustrate the rolling-window dynamic results of the SampEn algorithm within the period from May 2006 to February 2009 (two combined pre-GFC and GFC sub-periods), for selected European developed and emerging markets, respectively. The highest absolute value of the change in SampEn (reported in Table 2) was the main criterion for the choice.

As reported in Section 4.1 (Table 2), formal statistical analyses confirm that the basic statistics of entropy (measured by SampEn) decreased significantly during the GFC period but, in general, the results are not such homogenous as in the case of the COVID-19 pandemic period. This evidence can be observed in Figure A1–Figure A3.
